# Immune modulation therapy in the management of bortezomib-induced peripheral neuropathy

**DOI:** 10.1186/2162-3619-1-20

**Published:** 2012-08-15

**Authors:** Ashley Jeter, Yubin Kang

**Affiliations:** 1 Division of Hematology and Oncology, Department of Medicine, Medical University of South Carolina, Charleston, South Carolina, USA; 2 Hollings Cancer Center, 86 Jonathan Lucas Street, Room# HO307, Charleston, SC, 29425, USA

**Keywords:** Bortezomib, Peripheral neuropathy, Immune modulation, Multiple myeloma

## Abstract

Peripheral neuropathy (PN) is one of the most common side effects of bortezomib therapy. The majority of bortezomib-related PN is a sensory neuropathy of mild to moderate degree, and is reversible after dose reduction or discontinuation. However, occasionally bortezomib-induced neuropathy can be severe and affects motor and/or autonomic nerves, and may be mediated by immune process. The role of immune modulation therapy in the management of bortezomib-induced PN was not well established. Here, we reported a case of bortezomib-induced severe PN that responded well to plasma exchange and steroid treatment.

## Background

Bortezomib is a reversible proteasome inhibitor, and has been approved by the FDA for the treatment of multiple myeloma either in combination with other agents or as a single agent [1,2]. One of the common side effects associated with bortezomib therapy is PN [3-7]. The neuropathy may be caused by direct toxic effect of bortezomib or through an immunologically mediated process [3-7]. Here, we reported our experience in managing a severe bortezomib-related PN with immune modulation treatment.

### Case presentation

This patient is a 65 years old Caucasian male who was diagnosed with multiple myeloma in December 2010 based on an IgG kappa paraprotein (3 g/dl), 25% kappa light chain restricted- plasma cells in bone marrow biopsy, and the presence of anemia (hemoglobin 9 g/dl). His past medical history was significant for type II diabetes mellitus well controlled with Metformin and Sitagliptin. On December 30, 2010, he was started on therapy with single agent bortezomib given at 1.3 mg/m^2^ intravenously weekly. Dexamethasone was not given due to concern for hyperglycemia with his underlying diabetes. On January 20, 2011, lenalidomide was added at 10 mg a day two weeks on and one week off schedule. On February 15, 2011, during approximately the third cycle of bortezomib treatment, the patient started having severe pain in both legs extending from his thighs to his calves that he described as “toothache-like” pain as well as some mild paresthesias and muscle spasms. His chemotherapy was stopped on March 8, 2011. He also developed progressively worsening bilateral lower extremity weakness. Within one week, the patient was unable to walk. He could not move his legs and required the assistance of a wheelchair for mobility.

On physical examination: the cranial nerves were normal and extraocular muscles were intact. Manual motor test was performed and showed: Deltoid, biceps, triceps, and wrist extensor 5/5, finger extensor 4/5 bilaterally, first dorsal interosseous 3+/5 bilaterally, abductor digiti minimi 3/5 bilaterally, abductor pollicis brevis 4/5 bilaterally. Iliopsoas 4/5 right and 4-/5 left, quadriceps femoris 5/5 bilaterally, hamstrings 4/5 bilaterally, tibialis anterior 4-/5 in the right and 1/5 left. Sensory Examination: Decreased pin prick in distal gradient distribution to above ankles. Temperature sensation was intact. Vibratory sensation at toes was 1 bilaterally, at knees 4 bilaterally, and at right finger 5. Deep tendon reflexes were zero throughout. Magnetic resonance imaging of cervical, thoracic, and lumbosacral spine was normal. Cerebrospinal fluid showed normal cell count at 2 × 10^6^/L, but high protein level at 133 mg/dl. Nerve conduction study (NCS) and electromyogram (EMG) showed electrophysiological evidence of widespread severe sensory motor peripheral neuropathy with the predominantly axonal demyelination features. Serum anti-GM1, anti-NS6S, anti-MAG, anti-GQ1b, anti-Ho and anti-Yu antibodies were negative.

The patient underwent plasma exchange five times. After the second exchange, he was able to ambulate with a walker. However, his condition subsequently worsened. On May 31, 2011 prednisone 40 mg PO daily was initiated. Within one to two days after prednisone, his motor strength started to improve. Two months later, prednisone was gradually tapered and was completely off on September 9, 2011. At the time of discontinuation of prednisone, he was able to ambulate with a cane and currently his performance status is back to his status prior to bortezomib treatment. He is currently in complete remission from multiple myeloma.

## Conclusion

Bortezomib-induced PN occurs mainly in two forms: the first is a direct toxic neuropathy, which is late onset, much more common, and in general, not as rapidly progressive and severe. The second form is immune mediated PN [3,7], which is early onset, less common, characterized by prominent motor involvement, and responds to steroids and intravenous immunoglobulin. Our patient likely developed the second form of PN (i.e., immune mediated PN). Our patient responds well to plasma exchange and prednisone, and had nearly complete resolution of his PN.

Currently, there are published guidelines for the management of bortezomib-induced PN including how to grade PN, when and how to adjust bortezomib dose, and how to use adjunctive measures [6,8,9]. These guidelines should be followed and all patients who are receiving bortezomib should be closely monitored for the development of any sensory, motor, or autonomic neuropathy. In addition, immune therapy such as plasma exchange, IVIG and/or steroid should be considered if there is evidence of immune-mediated neuropathy such as unexplained worsening of neurological dysfunction despite bortezomib discontinuation, with prominent motor involvement, and CSF signs of inflammation [3,7,10]. The overall outcome of bortezomib-related, immune mediated, severe PN is still quite favorable with the potential of complete resolution of the neuropathy.

## Consent

Consents were obtained from the patient for publishing these data.

## Competing interests

The authors declared no competing financial interests.

## Authors’ contributions

Both Drs. Jeter and Kang participated in the care of the patient and wrote the manuscript.

## Authors’ information

Dr. Jeter is a hematology-oncology fellow at the Medical University of South Carolina. Dr. Kang is an assistant professor and attending physician at the Division of Hematology-Oncology, Medical University of South Carolina.
